# Draft genome sequence of a non-human primate-derived isolate of *Candida parapsilosis*

**DOI:** 10.1128/mra.00811-24

**Published:** 2024-12-10

**Authors:** Steve A. James, Aimee Parker, Catherine Purse, David J. Baker, Simon G. P. Funnell, Simon R. Carding

**Affiliations:** 1 Food, Microbiome and Health Programme, Quadram Institute Bioscience, Norwich, United Kingdom; 2 UK Health Security Agency, Porton Down, Salisbury, United Kingdom; 3 Norwich Medical School, University of East Anglia, Norwich, United Kingdom; University of Maryland School of Medicine, Baltimore, Maryland, USA

**Keywords:** *Candida parapsilosis*, fungal pathogen, gut mycobiome, non-human primate, cynomolgus macaque, genome sequence

## Abstract

*Candida parapsilosis* is a common human commensal and opportunistic fungal pathogen that is also found in non-human primates (NHPs). Here, we report the first draft sequence of *C. parapsilosis* NCYC 4418, a fecal isolate from an adult cynomolgus macaque.

## ANNOUNCEMENT

*Candida parapsilosis* is a dimorphic ascomycete yeast belonging to the *Lodderomyces* clade (1). With the capacity to form persistent biofilms, on both biotic and abiotic surfaces, it has become a major global cause of invasive candidiasis, most notably in preterm and low-birthweight neonates (2). While considered a commensal member of the human gastrointestinal tract and skin (3–5), *C. parapsilosis* is also found in non-human primates (NHPs) (6, 7). Here, we combined short- and long-read sequencing to obtain the genome sequence of *C. parapsilosis* NCYC 4418, a fecal isolate from a healthy adult cynomolgus macaque housed at a managed breeding colony (UK). Fecal homogenate, prepared in sterile phospate-buffered saline (PBS), was spread onto solid Sabouraud dextrose (SD) medium containing penicillin (25 U/mL) and streptomycin (25 U/mL) and was incubated for 2 d at 37°C. Colonies were picked and purified by three rounds of re-streaking on fresh SD agar. Colony isolate NCYC 4418 was identified as *C. parapsilosis* by ITS1 sequencing using primers ITS1F and ITS2 (8, 9). The ITS1 sequence was deposited in GenBank (accession number OZ120394.1).

Genomic DNA was extracted from a 10-mL stationary phase SD culture using a MasterPure yeast DNA purification kit (Cambio) with additional zymolyase and proteinase K treatment steps. Illumina short-read sequencing used a modified 20-fold dilution of DNA Prep (Flex) reagent and run on a NextSeq 500 sequencer, producing 12,162,590 paired-end 150-bp reads (~134× coverage). A Nanopore library was prepared from non-sheared DNA (not size selected), and sequencing was performed using a MinION sequencer (Oxford Nanopore Technologies, ONT), ligation sequencing SQK-LSK109 kit (ONT), and flow cell FLO-MIN106 R9.4.1 (ONT). This produced a total of 180,907 reads with a *N*_50_ of 17,699 bp (~58× coverage). Default parameters were used for all software unless otherwise specified. Base calling was performed using Guppy (ONT; v3.6.0) in a high-accuracy mode (model dna_r9.4.1_450bps_hac). Raw short- and long-read polishing was performed using fastp 0.23.2 (10).

*De novo* assembly of the Nanopore reads was performed using Flye 2.9.1 (11), and the Illumina short reads were used for contig error correction (polishing) using BWA v0.7.17.1 (12) and Pilon v1.24 (13). Assembly statistics were generated using QUAST v5.0.2 (14). The resulting assembly comprised 14 contigs, including four chromosome-sized contigs with telomeric termini (15). The total genome size was 13,056,352 bp, with a *N*_50_ of 2,100,473 bp and a G+C content of 38.70%. The largest contig was 3,026,409 bp and corresponded to chromosome 2 (16). A mitochondrial genome sequence, representing 94.7% of total length (16), was also recovered in three contigs. Genome completeness was estimated as 98.9% using BUSCO v5.5.0 (17) with the saccharomycete_odb10 data set. Augustus v3.2.3 (18) predicted 5,587 protein-coding genes using the *Candida albicans* training data set, and 105 tRNA genes (91 nuclear encoded) were detected using tRNAscan SE 2.0 (19). Sequencing coverage was assessed using SAMtools and SAMtools depth (20), with the primate isolate found to carry 10 copies of *ARR3*, a putative arsenate transporter-encoding gene frequently amplified in copy number in both human and environmental isolates (21).

## Data Availability

This whole-genome shotgun project has been deposited in DDJB/ENA/GenBank (BioProject number PRJEB77687, assembly accession number CAXMZS000000000.1). The version described in this paper is version 1. The raw reads are available in the NCBI Sequence Read Archive (SRA) under accession numbers SRR29413522 (ONT) and SRR29413523 (Illumina).
